# Lockdown and psychological stress in Wuhan, China

**DOI:** 10.1371/journal.pone.0274696

**Published:** 2023-04-07

**Authors:** Mengying Li, Wenjing Wang, Boya Zhu, Qi Chen, Yubin Zhang, Shuzhen Peng, Ling Zhang, Xiaodong Tan

**Affiliations:** 1 Department of Occupational and Environmental Health, School of Public Health, Wuhan University, Wuhan, China; 2 Wuchang Center for Disease Control and Prevention, Wuhan, China; 3 People’s Hospital of Huangpi, Wuhan, China; Institute for Advanced Sustainability Studies, GERMANY

## Abstract

**Background:**

Wuhan was the epicenter of the Coronavirus Disease 2019 (COVID-19), outbreak in China. We aimed at surveying the general public in China to better understand their levels of psychological state and its influencing factors after the Wuhan shutdown on January 23.

**Methods:**

The cross-sectional survey was conducted online and 4,701 respondents participated in this survey. Of them, 3,803 respondents were considered for final analysis. Data on subjective indicators of daily life changes were collected, and individual scores on changes in anxiety, depression, and stress were tested by 8-item, 11-item, and 6-item questionnaires, respectively.

**Results:**

Multivariable regression analyses showed that living in the rural areas, living in the other regions except Hubei, and higher education were independent correlates of less negative emotions. Besides, the level of attention, self-assessed infection risk, impact on the daily life and mental health help-seeking tended to be positively associated with the scores of anxiety, depression, and stress.

**Conclusions:**

City of residence, education, marital status, monthly income, level of attention, self-assessed infection risk, impact on daily life and mental health help-seeking were important correlates of anxiety, depression, and stress scores.

## Introduction

COVID-19, which had led to a national pandemic and rapid global transmission to many countries worldwide [1], and stringent public health measures have been implemented to curtail the spread of COVID-19. In China, the government had taken effective measures to prevent further dispersal, including lockdowns, closing entertainment venues, extending the Chinese New Year holidays, forcing people to wear masks in public, and limiting the number and frequency of outings per household [2]. The lockdown was an emergency measure, meaning that people should stay where they are and not allow anyone to enter or leave, which greatly reduces social activity. These measures had been proved to be a huge success in the fight against COVID-19 [3], but have also created economic and psychological burdens [4].

The outbreak itself and the measures taken to combat the epidemic could lead to widespread fear and panic, which might escalate into further negative psychological reactions, including psychological distress, depression, anxiety, and post-traumatic stress symptoms, as well as sleep disturbance, palpitations and suicidality [5–8]. With the closure of schools and businesses, the negative emotions experienced by individuals become more complicated [9]. One recent study pointed to an increase in psychological problems during this epidemic, including anxiety, depression, and stress [10]. A study of 3480 participants from Spain during an outbreak found that 21.6% had anxiety and 18.7% had depression [11]. Studies had shown that young people, women, low socioeconomic status, mental illness, and chronic health problems are risk factors for depression [12]. The COVID-19 pandemic led a serious impact on people ’s economic levels, which was also an important reason for the rise in depressive symptoms among adults [13]. At the same time, as most residents were restricted to their homes, they tended to face too much negative news at home every day, and those with higher education and those who pay more attention to the epidemic news show higher levels of anxiety and depression [14]. Furthermore, the pandemic also affected specific populations. A recent systematic review found that compared with the general population, the COVID-19 patients showed high levels of post-traumatic stress symptoms and higher depressive symptoms [15]. And two studies noted an increase in psychological problems during the epidemic and emphasized that we should pay attention to the mental health of specific groups, such as children, older adults, patients, and medical staffs [16–18].

The COVID-19 pandemic has also changed people’s lifestyles, which may affect mental health. A study in Italy suggested that during the COVID-19 lockdown, 48.6% of people felt weight gain, whereas a slight increased physical activity has been reported in 38.3% of respondents, and 15% of respondents turned to farmers or organic purchasing groups for fruit and vegetables [19]. Regular exercise may reduce depression and anxiety [20]. Evidence from a review showed that diet, physical activity, sleep, and mental stress levels have been altered during the pandemic. The decline of sleep quality also affects mental health [21]. Changes in dietary habits than usual and weight gain were observed. It also found that the prevalence of any kind of mental stress, especially anxiety, is the highest [22].

One of the results from the Indian epidemic period showed that with the increase in isolation time, the psychological status of the population gradually decreased, which may be due to the relatively few mental health interventions made by the government [23]. Therefore, the psychological status of people should be concerned in many aspects, as it is closely related to a healthy body and healthy life of population. However, there was no data available to assess the impact of the COVID-19 pandemic on the mental health of the general population in China one month after the Wuhan lockdown. Therefore, we conducted a survey to investigate the residents’ changes in life and psychological conditions. This might assist government agencies and healthcare professionals in safeguarding the psychological well-being of the community in the face of the COVID-19 outbreak expansion in China and different parts of the world.

## Materials and methods

### Participants

We conducted an online survey one month (February 20 to 24) after the shutdown of Wuhan (January 23) and Hubei Province (January 25) against the COVID-19 spread. Due to the peculiarities of the epidemic, face-to-face communication was not possible for this survey, so our questionnaires were conducted on social networks. We distributed a self-report questionnaire to the Chinese public through the WeChat platform. The questionnaire consisted of two parts, one was sociodemographic characteristics, including gender, age, place of residence, education level, marital status, occupation, whether to be quarantined, and whether to be diagnosed with COVID-19; and the other is the psychological status of the public.

And we received 4071 anonymous questionnaires in the investigation, covering 33 Chinese provinces and autonomous regions except for Taiwan. The inclusion criteria were as follows: 1. Male or female ages 15–85 years; 2. Participants must have the capacity to understand the study; 3. Participants must be fluent in Chinese; and 4. Participants currently live in China. And the exclusion criteria were as follows: 1. Serious neurological (specific or focal) disorders preventing full participation in the protocol; 2. logical responses in the questionnaire (e.g., selecting the same option consecutively; the results of similar choices vary widely).

After eliminating the invalid samples, 3803 (93.42%) valid questionnaires were finally obtained. This study was approved by the research ethics committees of Wuhan University (Approval No. 2020YF0064). Participants voluntarily responded to the anonymous survey. Written informed consent was obtained from all participants in the study, as well as that of minor’s parents or guardians. Based on the investigation of the psychological state after the disaster in China and after discussion with experts, the self-administered questionnaires were mainly divided into three parts.

### Self-reported changes in psychological status

In the study, **21 feelings-related items** were used to measure the changes in psychologic status, including “I feel nervous”, “I feel anxious”, “I feel scared”, “I feel tired and fatigued”, “I feel irritable and easily angry”, “I feel lonely”, “I feel sad and want to cry”, “I don’t want to talk to others”, “I think other people unfriendly to me”, “I have a hard time concentrating on work”, “I don’t sleep well”, “My total sleep time”, “I have a bad appetite”, “My weight”, “I feel like a failure”, “I am worried about the future”, “I feel strenuous to do things”, “I feel palpitations”, “I feel chest tightness, shortness of breath”, “I feel uneasy, unable to sit still”, “I feel muscle pain, limited mobility”.

We rated these items in a 5-point response format (“-2 = significantly decrease”, “-1 = decrease”, “0 = unchanged”, “1 = increase”, or “2 = significantly increase”). Sorting the items and calculating the scores can help analyze the changes in the residents’ psychological status in a more targeted manner. Therefore, a literature review and expert interview methods were used to construct the index system first. According to the literature [24–26], the 21 feeling items in the homemade questionnaires were classified into three categories: anxiety, depression, and stress.

The total scores were calculated by simple addition based on the extent of the feeling. A negative score indicated that the negative emotions of the participants decreased compared to the previous week; otherwise, a positive score indicated that the negative emotions increased. The higher the score, the worse the psychological condition was. **An additional file showed the questionnaire in more detail [see S1 File].** The reliability of the questionnaire was checked using Cronbach’s alpha, and the reliability coefficient was 0.958.

### Subjective indicators of changes in daily life

The status of the daily life of residents after the Wuhan shutdown was composed of the level of attention, self-assessed infection risk, the impact of daily life, self-perceived health status, mental health help-seeking and satisfaction with community work. The first 3 items were rated as “1 = decreased”, “2 = unchanged”, and “3 = increased”, while self-perceived health status was rated as “1 = good/very good”, “2 = average”, and “3 = bad/very bad”. Mental health help-seeking was rated as “1 = found and tried”, “2 = found but not tried”, “3 = not found yet”, “4 = not looked for”, and “5 = no need to adjust”, and satisfaction with community work was rated as “1 = satisfied”, “2 = general”, and “3 = unsatisfied”.

### Covariates

The following variables were used as covariates: gender (1 = male, 0 = female), age (1 = <30, 2 = 30–49, 3 = ≥50), education (1 = middle school or below, 2 = high school, 3 = college, 4 = master’s degree and above), place of residence (1 = urban, 2 = rural), current residence (1 = Wuhan, 2 = other cities of Hubei Province, 3 = other areas of China), marital status (1 = single, 2 = married, 3 = divorced or other), occupation (0 = nonmedical staff, 1 = medical staff), monthly income (yuan) (1 = <2000, 2 = 2000–5000, 3 = 5001–10,000, 4 = >10,000), number of cohabitants (1 = 0, 2 = 1, 3 = 2–3, 4 = ≥4), quarantine or not (0 = no, 1 = yes), and confirmed cases in personal network (0 = no, 1 = yes).

### Statistical analysis

Data were double-entered and cross-checked using Excel version 2019 (Microsoft Corp.; Redmond, USA), R software (R version 3.6.2) was used for data cleaning, SPSS software (SPSS, version 25.0) was used to conduct corresponding statistical analysis, and a two-sided *p* value less than 0.05 was considered statistically significant.

To identify the determinants of participants’ psychological feelings, we first examined the effects of their characteristics on changes in anxiety, depression, and stress scores with one-way analysis of variance (ANOVA) or the nonparametric Kruskal-Wallis test for categorical variables, depending on the distribution of the variables. The statistically significant variables were then allowed to enter the multiple linear regression model, and dummy variables were created when appropriate. Dichotomous variables were explored for the regression analysis to simplify the relationships. A series of multiple linear regression analyses (stepwise method) were explored to investigate the independent associations between sociodemographic variables, subjective indicators of changes in daily life and summary scores, including anxiety, depression, and stress scores, after checking the assumptions of the distribution and the independence of the residuals, as well as multicollinearity. Normality was assessed by visual inspection of the P-P plot. Linearity and homoscedasticity were investigated by visual inspection of the plot of the predicted values and standardized residuals. A variance inflation factor (VIF) of greater than 10 was used to identify possible multicollinearity among independent variables.

## Results

**Table 1** described the sociodemographic distribution of the study participants. During the study period, a total of 3803 residents participated in the study (34.4% male and 63.6% female), 432 (11.44%) people were in quarantine, and 718 (18.9%) said that people they knew were diagnosed with COVID-19.

**Table 1:** Factors affecting anxiety, depression, and stress in the survey (n = 3803; X±S; n/%).

Variable	N (%)	Anxiety	t/F	P	Depression	t/F	P	Stress	t/F	P
Gender			0.038	0.845		0.014	0.907		0.034	0.853
Male	1307(34.4)	-2.96±5.61			-2.73±7.22			-2.16±4.14		
Female	2496(65.6)	-2.99±5.36			-2.70±6.91			-2.18±3.94		
Age			0.440	0.644		0.281	0.755		0.016	0.984
<30	1150(30.2)	-3.02±5.70			-2.65±7.58			-2.16±4.24		
30–49	1986(52.2)	-3.02±5.43			-2.79±6.93			-2.18±3.98		
≥50	667(17.5)	-2.80±5.06			-2.58±6.23			-2.16±3.68		
Place of living			40.493	<0.001		32.425	<0.001		29.019	<0.001
Urban	2953(77.6)	-2.68±5.23			-2.36±6.67			-1.98±3.84		
Rural	850(22.4)	-4.02±6.03			-3.91±7.99			-2.82±4.48		
City of residence			28.854	<0.001		17.346	<0.001		29.307	<0.001
Wuhan	1196(31.4)	-2.04±5.17			-1.73±6.45			0.33±1.71		
Other cities of Hubei Province	923(24.3)	-3.09±5.70			-3.01±7.29			-0.05±1.93		
Other areas of China	1684(44.3)	-3.59±5.41			-3.24±7.19			-0.21±1.81		
Education			1.786	0.148		1.520	0.207		1.879	0.131
Middle school or below	130(3.4)	-3.05±5.76			-2.76±6.97			-2.22±4.37		
High school	489(12.9)	-3.21±5.91			-2.96±7.63			-2.24±4.35		
College	2413(63.4)	-3.05±5.47			-2.81±7.10			-2.25±4.03		
Master’s degree and above	771(20.3)	-2.59±4.98			-2.71±7.02			-1.87±3.63		
Marital status			0.532	0.588		0.812	0.444		0.241	0.786
Single	1101(29.0)	-2.99±5.62			-2.55±7.56			-2.15±4.23		
Married	2530(66.5)	-3.01±5.38			-2.81±6.78			-2.19±3.91		
Divorced/Other	172(4.5)	-2.56±5.33			-2.31±6.86			-1.99±4.08		
Occupation			0.203	0.652		1.151	0.283		0.032	0.859
Medical staff	1015(26.7)	-2.91±5.51			-2.91±6.98			-2.19±4.08		
Nonmedical staff	2788(73.3)	-3.00±5.42			-2.64±7.03			-2.16±3.99		
Monthly income (yuan)			3.171	0.023		3.914	0.008		2.804	0.038
<2000	779(20.5)	-3.48±5.86			-3.31±7.80			-2.50±4.38		
2000–5000	1143(30.1)	-2.87±5.59			-2.65±6.97			-2.16±4.08		
5001–10,000	1030(27.1)	-2.96±5.27			-2.79±6.94			-2.14±3.87		
>10,000	851(22.4)	-2.69±5.03			-2.14±6.36			-1.93±3.71		
Number of cohabitants			0.943	0.419		1.225	0.299		0.488	0.691
0	216(5.7)	-2.40±5.93			-1.84±7.64			-1.88±4.29		
1	455(12.0)	-2.97±5.61			-2.88±7.12			-2.26±4.12		
2–3	1921(50.5)	-2.98±5.37			-2.74±6.93			-2.17±3.94		
≥4	1211(31.8)	-3.08±5.41			-2.75±7.00			-2.19±4.02		
Quarantine or not			1.593	0.207		2.158	0.142		3.245	0.072
Yes	432(11.4)	-2.67±5.48			-2.24±7.15			-1.84±4.05		
No	3371(88.6)	-3.02±5.44			-2.77±7.00			-2.21±4.00		
Confirmed infected in personal network			36.915	<0.001		28.420	<0.001		32.041	<0.001
Yes	718(18.9)	-1.87±5.20			-1.46±6.40			-1.41±3.77		
No	3085(81.1)	-3.24±5.47			-3.00±7.13			-2.35±4.04		
Level of attention			29.968	<0.001		11.985	<0.001		27.088	<0.001
Decreased	1503(39.5)	-3.77±5.34			-3.23±7.20			-2.72±3.98		
Unchanged	1148(30.2)	-2.17±4.85			-1.90±6.21			-1.59±3.53		
Increased	1152(30.3)	-2.76±5.98			-2.83±7.46			-2.04±4.39		
Self-assessed infection risk			166.344	<0.001		102.190	<0.001		153.937	<0.001
Decreased	2738(72.0)	-3.92±5.56			-3.69±7.38			-2.84±4.10		
Unchanged	849(22.3)	-0.87±4.08			-0.38±5.07			-0.66±3.04		
Increased	216(5.7)	0.65±4.81			0.57±5.72			0.38±3.49		
Impact on daily life			138.122	<0.001		141.736	<0.001		132.702	<0.001
Decreased	873(23.0)	-5.42±6.14			-5.95±8.38			-3.93±4.52		
Unchanged	1701(44.7)	-2.73±4.66			-2.27±5.99			-1.99±3.43		
Increased	1229(32.3)	-1.60±5.36			-1.01±6.51			-1.17±3.96		
Self-perceived health status			44.227	<0.001		32.554	<0.001		36.854	<0.001
Good/Very good	3509(92.3)	-3.22±5.40			-2.97±7.01			-2.33±4.00		
Average	272(7.2)	-0.22±5.00			0.28±6.08			-0.27±3.56		
Poor/Very poor	22(0.5)	0.73±7.19			1.95±8.58			-0.14±4.24		
Mental health help-seeking			34.648	<0.001		35.967	<0.001		32.557	<0.001
Found and tried	263(6.9)	-4.33±5.80			-4.63±7.97			-0.45±1.92		
Found but not tried	79(2.1)	-2.91±5.69			-1.43±6.72			0.05±1.71		
Not found yet	82(2.2)	0.13±5.59			0.72±6.76			1.05±1.94		
Not looked for	1169(30.7)	-1.73±5.21			-1.04±6.52			0.44±1.78		
No need to adjust	2210(59.1)	-3.60±5.35			-3.54±6.96			-0.22±1.77		
Satisfaction with community work			29.250	<0.001		22.894	<0.001		29.421	<0.001
Satisfied	3140(82.6)	-3.29±5.50			-3.06±7.15			-2.40±4.04		
General	441(11.6)	-1.62±4.87			-1.13±6.02			-1.20±3.71		
Dissatisfied	222(5.8)	-1.33±5.09			-0.89±6.27			-0.90±3.58		

Multivariable analyses were then performed to identify these variables with a significantly independent impact on the changes in psychological status **(Table 2)**. The scores of anxiety, depression, and stress were dependent variables, and the independent variables of the models were age (≥50 as reference), gender (male as reference), place of living (urban as reference), city of residence (other areas in China as reference), education (middle school or below as reference), marital status (single as reference), occupation (medical staff as reference), monthly income (<2,000 yuan as reference), number of cohabitants (0 as reference), quarantine or not (yes as reference), confirmed infection in the personal network (yes as reference), level of attention (increased as reference), self-assessed infection risk (increased as reference), mental health help-seeking (not found yet as reference) and satisfaction with community work (dissatisfied as reference). The anxiety (B = -1.27, 95% CI = -1.71 to -0.82), depression (B = -1.47, 95% CI = -2.06 to -0.88), and stress (B = -0.79, 95% CI = -1.13 to -0.46) scores of people in rural areas were lower than those of people in urban areas. In addition, compared with people living in Hubei Province, people in other parts of China have lower anxiety and stress scores. In terms of education, the mental health status of people with higher education is better than people with middle school education and below. Married persons had lower anxiety, depression, and stress scores than single persons did; that is, their mental health was relatively better. People with monthly incomes above 10,000 yuan have higher anxiety, depression, and stress scores than those with monthly incomes below 2,000 yuan. Taking people who had sought mental health help but not yet as a reference, people who had tried to adjust their mental state in some way and those who thought they did not need to adjust their mental state had lower scores of anxiety, depression, and stress. In addition, people who had received psychological help had lower anxiety and stress scores. Gender, age, occupation, confirmed infection in personal networks and satisfaction with community work did not appear to be significant correlates of anxiety, depression, or stress scores.

**Table 2:** Multivariate regression model for factors associated with anxiety, depression, and stress.

Variables	Categories	Anxiety	Depression	Stress
Gender	Male	Reference	Reference	Reference
	Female	-0.10(-0.44 to 0.25)	-0.10(-0.56 to 0.35)	-0.07(-0.32 to 0.19)
Age	≥50	Reference	Reference	Reference
	<30	0.39(-0.33 to 1.11)	0.48(-0.46 to 1.43)	0.45(-0.08 to 0.99)
	30–49	0.01(-0.45 to 0.46)	-0.06(-0.65 to 0.53)	0.14(-0.20 to 0.47)
Place of living	Urban	Reference	Reference	Reference
	Rural	-1.27*(-1.71 to -0.82)	-1.47* (-2.06 to -0.88)	-0.79*(-1.13 to -0.46)
City of residence	Other provinces and cities	Reference	Reference	Reference
	Wuhan, Hubei Province	0.63* (0.20 to 1.06)	0.34(-0.22 to 0.90)	0.51* (0.20 to 0.83)
	Other cities in Hubei Province	0.58* (0.13 to 1.03)	0.43(-0.17 to 1.02)	0.39* (0.05 to 0.72)
Education	Middle school or below	Reference	Reference	Reference
	High school	-1.01* (-1.99 to -0.03)	-1.21(-2.50 to 0.07)	-0.60(-1.33 to 0.12)
	College	-1.38* (-2.30 to -0.45)	-1.90* (-3.12 to -0.69)	-1.03* (-1.71 to -0.34)
	Master’s degree and above	-1.13* (-2.14 to -0.13)	-1.68* (-2.99 to -0.37)	-0.82* (-1.56 to -0.08)
Marital status	Single	Reference	Reference	Reference
	Married	-0.67* (-1.30 to -0.05)	-1.14* (-1.96 to -0.33)	-0.47* (-0.93 to 0.00)
	Divorced/Other	-0.41(-1.38 to 0.56)	-0.88(-2.14 to 0.39)	-0.36(-1.08 to 0.35)
Occupation	Medical staff	Reference	Reference	Reference
	Nonmedical staff	-0.25(-0.65 to 0.15)	-0.51(-1.03 to 0.02)	-0.24(-0.53 to 0.05)
Monthly income (yuan)	<2000	Reference	Reference	Reference
	2000–5000	0.29(-0.26 to 0.84)	0.66(-0.06 to 1.38)	0.21(-0.20 to 0.61)
	5001–10,000	0.27(-0.33 to 0.86)	0.56(-0.22 to 1.34)	0.30(-0.14 to 0.74)
	>10,000	0.73* (0.08 to 1.38)	1.45* (0.60 to 2.30)	0.65* (0.17 to 1.13)
Number of cohabitants	0	Reference	Reference	Reference
	1	-0.23(-1.05 to 0.59)	-0.84(-1.91 to 0.23)	-0.15(-0.76 to 0.45)
	2~3	0.01(-0.72 to 0.74)	-0.34(-1.30 to 0.61)	0.11(-0.43 to 0.65)
	≥4	0.12(-0.64 to 0.88)	-0.11(-1.11 to 0.88)	0.20(-0.37 to 0.76)
Quarantine or not	Yes	Reference	Reference	Reference
	No	0.30(-0.21 to 0.81)	0.43(-0.23 to 1.10)	0.32(-0.05 to 0.70)
Confirmed infected in personal network	Yes	Reference	Reference	Reference
	No	-0.32(-0.79 to 0.14)	-0.52(-1.13 to 0.09)	-0.18(-0.52 to 0.16)
Level of attention	Increased	Reference	Reference	Reference
	Reduced	-0.95* (-1.35 to -0.54)	-0.39(-0.92 to 0.15)	-0.67* (-0.97 to -0.36)
	Unchanged	0.24(-0.19 to 0.67)	0.53(-0.02 to 1.09)	0.19(-0.13 to 0.50)
Self-assessed infection risk	Increased	Reference	Reference	Reference
	Reduced	-3.47* (-4.18 to -2.76)	-2.88* (-3.81 to -1.95)	-2.44* (-2.96 to -1.91)
	Unchanged	-1.17* (-1.93 to -0.42)	-0.60(-1.59 to 0.40)	-0.81* (-1.37 to -0.25)
Impact on daily life	Increased	Reference	Reference	Reference
	Reduced	-2.86* (-3.32 to -2.41)	-4.06* (-4.66 to -3.47)	-2.10* (-2.43 to -1.76)
	Unchanged	-0.81* (-1.19 to -0.43)	-1.02* (-1.52 to -0.52)	-0.60* (-0.89 to -0.32)
Mental health help-seeking	Not found yet	Reference	Reference	Reference
	Found and tried	-3.29* (-4.53 to -2.05)	-4.05* (-5.68 to -2.42)	-2.22* (-3.14 to -1.29)
	Found but not tried	-2.22* (-3.76 to -0.67)	-1.09(-3.12 to 0.93)	-1.55* (-2.69 to -0.40)
	Not looked for	-1.34* (-2.47 to -0.22)	-1.18(-2.65 to 0.29)	-0.79(-1.62 to 0.04)
	No need to adjust	-2.60* (-3.71 to -1.50)	-3.02* (-4.47 to -1.57)	-1.71* (-2.53 to -0.89)
Satisfaction with community work	Dissatisfied	Reference	Reference	Reference
	Satisfied	-0.40(-1.10 to 0.31)	-0.48(-1.40 to 0.44)	-0.39(-0.91 to 0.13)
	General	0.18(-0.64 to 0.99)	0.12(-0.94 to 1.18)	0.01(-0.59 to 0.61)

## Discussion

Since the COVID-19 outbreak sparked a global public health crisis by spreading across China and other countries, various mandatory precautions have been taken by governments and individuals [27]. Studying residents’ psychological conditions at this time point could benefit government agencies and healthcare professionals to protect the mental health of the community as their measures will be conducted in a more humanized way.

In multivariable analyses, we found that urban residents were more likely to gain anxiety, depression and stress than rural counterparts. In densely populated urban areas, well-planned efficient public transportation systems can facilitate residents’ travel [28]. The disruption of daily life and the absence of entertainment or recreation made it impossible to release the excess inner pressure of urban residents. A more important reason is that due to the high density of the urban population and the greater mobility of people than rural areas, the risk of disease infection is greater. High population density increases people’s exposure to infectious diseases [29], which may lead to increased negative emotions among urban residents. Besides, people who were married reported greater mental health status, indicating that family support is of great importance [30], especially in coping with the stress caused by the COVID-19 pandemic. Some studies documented that low-income groups are more likely to suffer from depression and anxiety [31]. In Hubei Province, Li et al. have found that workers with income losses during the COVID-19 have a high risk of developing unfavorable mental health symptoms [32]. However, our study showed that the epidemic had a greater impact on high-income groups; people with monthly incomes above 10,000 have higher anxiety, depression, and stress scores than those with monthly incomes below 2,000, and their concerns about delays in working hours and subsequent deprivation of expected income may explain the high level of stress [33].

Another noteworthy finding of this study is that the subjective indicators of changes in daily life played an important role in the scores of people’s anxiety, depression and stress. Our study found that the greater the level of attention to COVID-19, the greater the negative emotions, which is in agreement with previous research [34]. And a lack of editorial control in social media can also bring incorrect information, fake news, misconception, and rumor, which are possibly intensifying psychological fear and rumination about contracting disease with an effect on behavior and social interactions [35]. Therefore, the content of health information provided during the epidemic needs to be based on evidence to avoid adverse psychological reactions.

Our findings also revealed that the level of self-assessed infection risk also influenced participants’ mental state. Anxiety, depression and stress outcomes were elevated with the increase in self-assessed infection risk. This may have resulted from actual conditions; respondents received signals from the surrounding environment and are supposed to make corresponding assessments of their risk of infection. The respondents who felt severely affected by the lockdown exhibited more obvious anxiety, depression, and stress than the rest of them, which indicates that guaranteeing day-to-day lives for residents will be beneficial for mental health [36].

In addition, people who had tried to adjust their mental state in some way and those who thought they did not need to adjust their mental state had lower scores of anxiety, depression, and stress. This is consistent with the previous result: the use of positive coping strategies was found to be associated with lower depressive symptoms and with less distress during the COVID-19 pandemic [37, 38]. During the COVID-19 epidemic, online mental health services have become the mainstream mental health services, including online cognitive behavioral therapy for depression, anxiety, and insomnia (e.g., on WeChat) [39]. Studies have shown that active coping and social support could be beneficial for dealing with a decrease in mental health due to the COVID-19 pandemic [40]. Therefore, for people with psychological problems, it is also a good choice to seek help from professionals on the Internet.

Not only does this study supplement knowledge of the psychological status of residents during the lockdown period, but it also helps to clarify the groups of people who are more likely to experience negative emotions when the disease is epidemic, which is especially helpful for China and other countries. Even so, it has some limitations. It is limited by its sample size, which is insufficient. And a cause-effect relationship cannot be established due to the inherent nature of cross-sectional design. Besides, in the data collection process, sources of bias include the potential selection bias of respondents. Because respondents were asked if they were willing to participate in the survey, resulting in volunteer bias and may not represent the general population. Some unknown and omitted confounding factors may exist in this study, instrumental variable analysis was used to control these confounding factors.

## Conclusions

The life and psychological state of the urban population had produced negative changes after the Wuhan shutdown on January 23. City of residence, education, marital status, monthly income, level of attention, self-assessed infection risk, the impact of daily life and mental health help-seeking are important correlates of the scores of anxiety, depression, and stress. At present, China has achieved great success in the fight against epidemics, but the epidemic situation in some parts of the world has not improved. Most residents are still in a state of quarantine at home. Awareness of these relevant factors could help the government and related personnel prevent more severe psychological trauma in the later period.

## Supporting information

S1 Dataset (ZIP)

S1 File (DOCX)
