# Supplementary material for: Lockdown and psychological stress in Wuhan, China
Source: PLoS One. 2023 Apr 7;18(4):e0274696. doi: 10.1371/journal.pone.0274696 (PMC10081786; doi:10.1371/journal.pone.0274696)
Supplement: S1 File — (DOCX) [file pone.0274696.s002.docx]

**Questionnaire of changes in psychological status**

*Scale:*

-2=significantly decrease

-1=decrease

0=unchanged

1=increase

2=significantly increase

*Please check the box to the right of each question which best describes your experience* ***compared to last week.***

| 1. I feel nervous. | □-2 □-1 □0 □1 □2 |
| --- | --- |
| 2. I feel anxious. | □-2 □-1 □0 □1 □2 |
| 3. I feel scared. | □-2 □-1 □0 □1 □2 |
| 4. I feel tired and fatigued. | □-2 □-1 □0 □1 □2 |
| 5. I feel irritable and easily angry. | □-2 □-1 □0 □1 □2 |
| 6. I feel lonely. | □-2 □-1 □0 □1 □2 |
| 7. I feel sad and want to cry. | □-2 □-1 □0 □1 □2 |
| 8. I don't want to talk to others. | □-2 □-1 □0 □1 □2 |
| 9. I think other people unfriendly to me | □-2 □-1 □0 □1 □2 |
| 10. I have a hard time concentrating on work | □-2 □-1 □0 □1 □2 |
| 11. I don't sleep well | □-2 □-1 □0 □1 □2 |
| 12. My total sleep time | □-2 □-1 □0 □1 □2 |
| 13. I have a bad appetite | □-2 □-1 □0 □1 □2 |
| 14. My weight | □-2 □-1 □0 □1 □2 |
| 15. I feel like a failure. | □-2 □-1 □0 □1 □2 |
| 16. I am worried about the future. | □-2 □-1 □0 □1 □2 |
| 17. I feel strenuous to do things | □-2 □-1 □0 □1 □2 |
| 18. I feel palpitations | □-2 □-1 □0 □1 □2 |
| 19. I feel chest tightness, shortness of breath. | □-2 □-1 □0 □1 □2 |
| 20. I feel uneasy, unable to sit still. | □-2 □-1 □0 □1 □2 |
| 21. I feel muscle pain, limited mobility. | □-2 □-1 □0 □1 □2 |

Anxiety Score: Calculated by summing the individual scores to questions 1-4,11,12,18,21.

Depression Score: Calculated by summing the individual scores to questions 6-8,10-17.

Stress Score: Calculated by summing the individual scores to questions 1,2,5,11-13.

• A positive score indicates that the sentiment has increased compared to last week.

• A negative score indicates that the sentiment has decreased compared to last week.

• The larger the absolute value, the more the emotion changes.

**References Cited:**

1. Crawford JR, Henry JD. The Depression Anxiety Stress Scales (DASS): Normative data and latent structure in a large non-clinical sample. British Journal of Clinical Psychology 2003; 42(2): 111-31.

2. Enns MW, Cox BJ, Parker JD, Guertin JE. Confirmatory factor analysis of the Beck Anxiety and Depression Inventories in patients with major depression. Journal of affective disorders 1998; 47(1-3): 195-200.

3. Watson D, Clark LA, Tellegen A. Development and validation of brief measures of positive and negative affect: The PANAS scales. Journal of Personality and Social Psychology 1988; 54(6): 1063-70.

4. Beck AT, Epstein N, Brown G, Steer RA. An inventory for measuring clinical anxiety: Psychometric properties. Journal of Consulting and Clinical Psychology 1988; 56(6): 893-7.

5. Zigmond AS, Snaith RP. The hospital anxiety and depression scale. Acta Psychiatr Scand 1983; 67(6): 361-70.

6. Zung WWK. A Rating Instrument For Anxiety Disorders. Psychosomatics 1971; 12(6): 371-9.

7. Hamilton M. The assessment of anxiety states by rating. Br J Med Psychol 1959; 32(1): 50-5.

8. Derogatis LR, Lipman RS, Covi L. SCL-90: an outpatient psychiatric rating scale--preliminary report. 1973; 9(1): 13-28.
