# Supplementary material for: Lockdown and psychological stress in Wuhan, China
Source: PLoS One. 2023 Apr 7;18(4):e0274696. doi: 10.1371/journal.pone.0274696 (PMC10081786; doi:10.1371/journal.pone.0274696)
Supplement: S1 Dataset — (ZIP) [file pone.0274696.s001.zip › Variable assignment.doc]

**第二次调查**

number：序号

submit：提交时间

time:做问卷所用时间

source：来源

source_detail：来源细节

IP

province_IP：问卷来源省份

city_IP：问卷来源城市

截至目前，您本人及亲朋好友是否被确诊感染新型冠状病毒：

infected1：0：无 1：本人

infected2：0：无 1：家人或亲戚

infected3：0：无 1：朋友

infected4：0：无 1：认识的其他人

infected5：0：无 1：没有确诊者

frequency：最近一周，您对于新冠病毒肺炎疫情的关注频率（1：减少很多 2：减少了些 3：无明显变化 4：增加了些 5：增加很多）

您所在的社区，目前采取了哪些抗冠措施：

measure1：0：无 1：封锁社区，实行进出管控

measure2：0：无 1：进行社区保洁消毒工作

measure3：0：无 1：暂停人员聚集活动

measure4：0：无 1：大力宣传教育

measure5：0：无 1：逐一入户排查

measure6：0：无 1：对重点人群实行管控、隔离

measure7：0：无 1：统一发放口罩、消毒液等防护用品

measure8：0：无 1：统一采购、发放生活必需品

measure9：0：无 1：什么都没做

work_strength：您觉得您所在社区目前抗疫工作力度如何（1：非常满意，很到位 2：比较满意，还可以更好 3：一般，没什么感觉 4：不太满意，力度不够 5：很不满意，完全不作为）

infection_risk：对比2020年1月23日（武汉“封城”）前，您认为您个人近几日感染新型冠状病毒的风险（1：减少很多 2：减少了些 3：一样 4：增加了些 5：增加很多）

health_condition：您目前的总体健康状况如何？（1：非常健康 2：比较健康 3：一般 4：不健康，但能自理 5：不健康，生活不能自理）

目前为止，您采取过哪些措施保护自己远离新型冠状病毒传染：

protection1：0：无 1：出门佩戴口罩

protection2：0：无 1：不参加聚会或串门，尽量少去人多且封闭的场所

protection3：0：无 1：咳嗽或打喷嚏时用纸巾掩住口鼻，不用脏手触摸眼睛、鼻或口

protection4：0：无 1：保持手卫生，正确及时洗手

protection5：0：无 1：合理饮食、适量运动等，增强自身免疫力

protection6：0：无 1：密切关注身体状况，每天测量体温等

protection7：0：无 1：居室经常通风换气并保持整洁卫生

protection8：0：无 1：尚未采取任何防护措施

您觉得此次新冠肺炎疫情对自己造成影响的方面是：

influence1：0：无 1：衣食住行等生活方式

influence2：0：无 1：对生活的看法、态度

influence3：0：无 1：人际关系

influence4：0：无 1：社交模式

influence5：0：无 1：身体健康

influence6：0：无 1：心理健康

influence7：0：无 1：以后的规划

influence8：0：无 1：办公/学习方式

influence9：0：无 1：其他

influence10：0：无 1：没有影响

influence_strength：对比上一周，您认为此次新冠肺炎疫情对自己近几日的生活影响（1：完全没有增加 2：几乎没有增加 3：基本没有变化 4：增加了些 5：增加很多）

您通过何种途径了解新型冠状病毒感染的肺炎相关的信息：

pathway1：0：无 1：各级政府部门的通告

pathway2：0：无 1：央视新闻联播、人民日报等各级官方媒体的消息

pathway3：0：无 1：新浪等国内非官方媒体的消息

pathway4：0：无 1：BBC等各类境外媒体的消息

pathway5：0：无 1：微信公众号、微博、知乎等自媒体传播的消息

pathway6：0：无 1：社区宣传、村委宣传

pathway7：0：无 1：亲人、朋友间传播的消息

pathway8：0：无 1：其他

attitude_hot：您对网上疫情相关热点话题或突发事件的态度是（1：非常关注，主动了解，并积极参与其中（如发帖、评论、转发）、与周围人讨论 2：比较关注，主动了解，但不转发评论或与人讨论 3：一般关注，听说后才会去看下相关报道 4：不太关注，别人说什么就是什么 5：完全不想知道）

attitude_rumour：疫情期间，您对未经证实的网络热点议题的态度是（1：不加判别，完全相信 2：理性对待，并选择性进行求证 3：不关心，与我无关就不在意 4：完全不信，只相信官方媒体报道的消息）

对比上一周，您这周的感受：（1：减少很多 2：减少了些 3：无明显变化 4：增加了些 5：增加很多）

feeling17：我感到紧张

feeling18：我感到焦虑

feeling19：我感到害怕、恐惧

feeling4：我感到疲倦、乏力

feeling5：我感到暴躁、容易生气

feeling6：我感到孤独

feeling2：我感到悲伤、想哭

feeling20：我不想跟别人说话

feeling21：我觉得其他人对我不友善

feeling22：我很难集中精神工作

Feeling23：我睡眠不安宁

feeling8：我的总体睡眠时间

feeling24：我不想吃东西，胃口很差

feeling10：我的体重

feeling25：我觉得自己一生很失败

feeling26：我对未来感到担忧

feeling27：我做事情感到吃力

feeling13：我感到心悸

feeling14：我感到胸闷、呼吸困难

feeling15：我感到不安、无法静坐

feeling16：我感到肌肉酸痛、活动受限

adjustment：自从新型冠状病毒爆发以来，您是否寻求过心理健康帮助（进行心理咨询等）或尝试其他方法进行自我心理状态调整？（1：是，寻找到并尝试了 2：寻找到，还未尝试 3：寻找了，还未寻找到 4：未寻找，未尝试 5：觉得不需要调整）

您目前主要的减压方式？

decompress1：0：无 1：看电视

decompress2：0：无 1：与家人闲聊

decompress3：0：无 1：棋牌/麻将

decompress4：0：无 1：看书（包括电子阅读）、学习

decompress5：0：无 1：QQ、微信等社交软件

decompress6：0：无 1：微博、知乎、论坛等

decompress11：0：无 1：听歌

decompress8：0：无 1：运动

decompress9：0：无 1：吃零食（三餐之外的都算，包括水果）

decompress12：0：无 1：电子游戏

decompress13：0：无 1：与宠物玩耍

decompress7：0：无 1：网购

decompress14：0：无 1：手机看抖音等短视频

decompress15：0：无 1：发呆、冥想或者睡觉

decompress16：0：无 1：其他

area：所在地区属于（1：城区 2：农村）

age：您的年龄（1:18岁及以下 2:19-29岁 3:30-39岁 4:40-49岁 5：50-59岁 6:60岁及以上）

sex：您的性别（1：男 2：女）

education：您的受教育程度（1：小学及以下 2：初中 3：高中/中专 4：大专/本科 5：硕士及以上）

marriage：您的婚姻状况（1：未婚 2：已婚 3：离婚 4：其他）

occupation：您的职业（1：各级政府部门、企事业单位、党政机关和公众团体的工作人员 2：医务人员 3：其他专业技术人员（教师、工程技术人员、作家等专业人员） 4：职员（从事一般性事务工作的人员） 5：第三产业服务人员 6：产业工人 7：从事农林牧渔业的劳动者 8：私营企业主、个体经营者 9：学生 10：无业/待业人员 11：离退休人员 12：其他 13：保险行业从业人员）

obode：常住（工作/学习）所在地（1：湖北省武汉市 2：湖北省其他城市 3：其他省市 4：国外）

condition：您现在的工作/学习状态是（1：返程复工 2：线上办公/学习 3：仍在假期 4：无业/待业状态 5：一直在上班）

income：您的经济状况（1：2000以下 2：2000-5000 3：5001-10000 4：10001-15000 5：15000以上）

cohabitant：您现在跟几个人一起居住（1：0人（独自居住） 2：1人 3：2-3人 4：4人及以上）

quarantine_or_not：您目前是否处在隔离状态（1：否 2：是（居家隔离） 3：是（定点医院隔离） 4：是（方舱医院隔离） 5：是（酒店隔离） 6：是（其他））

reason：您被隔离的原因是（-3：您目前是否处在隔离状态问题选择“否” 1：确诊 2：疑似病例 3：有发热等症状 4：密切接触者 5：一般接触者（包括因返工等原因迁移后居家观察14日））

quarantine_time：您目前已经隔离几天（-3：您目前是否处在隔离状态问题选择“否” 1：未隔离 2：1-3天 3：4-7天 4：7-11天 5：12-14天 6：14天以上）
